# Weighted Gene Co-Expression Network Analysis and Treatment Strategies of Tumor Recurrence-Associated Hub Genes in Lung Adenocarcinoma

**DOI:** 10.3389/fgene.2021.756235

**Published:** 2021-11-16

**Authors:** Zhengze Shen, Shengwei Liu, Jie Liu, Jingdong Liu, Caoyuan Yao

**Affiliations:** ^1^ Yongchuan Hospital of Chongqing Medical University, Chongqing, China; ^2^ JiangJin Central Hosptial of Chongqing, Chongqing, China; ^3^ Department of Pharmacy, First People’s Hospital of Chongqing Liangjiang New District, Chongqing, China

**Keywords:** lung adenocarcinoma, tumor recurrence, weighted gene co-expression network analysis, hub genes, transcription factor

## Abstract

Despite the recent progress of lung adenocarcinoma (LUAD) therapy, tumor recurrence remained to be a challenging factor that impedes the effectiveness of treatment. The objective of the present study was to predict the hub genes affecting LUAD recurrence via weighted gene co-expression network analysis (WGCNA). Microarray samples from LUAD dataset of GSE32863 were analyzed, and the modules with the highest correlation to tumor recurrence were selected. Functional enrichment analysis was conducted, followed by establishment of a protein–protein interaction (PPI) network. Subsequently, hub genes were identified by overall survival analyses and further validated by evaluation of expression in both myeloid populations and tissue samples of LUAD. Gene set enrichment analysis (GSEA) was then carried out, and construction of transcription factors (TF)–hub gene and drug–hub gene interaction network was also achieved. A total of eight hub genes (*ACTR3*, *ARPC5*, *RAB13*, *HNRNPK*, *PA2G4*, *WDR12*, *SRSF1*, and *NOP58*) were finally identified to be closely correlated with LUAD recurrence. In addition, TFs that regulate hub genes have been predicted, including MYC, PML, and YY1. Finally, drugs including arsenic trioxide, cisplatin, Jinfukang, and sunitinib were mined for the treatment of the eight hub genes. In conclusion, our study may facilitate the invention of targeted therapeutic drugs and shed light on the understanding of the mechanism for LUAD recurrence.

## Introduction

As one of the most frequently diagnosed and severe tumors worldwide, lung cancer was estimated to cause almost one-quarter of all cancer deaths in 2021 (Siegel et al., 2021). Lung adenocarcinoma (LUAD) is the most common type of lung cancer, which accounts for more than 40% of lung cancer incidence (Shi et al., 2016). Although the resection of early-stage LUAD remains to be the best treatment option, over 30% of LUAD patients develop recurrence and finally lead to dismal outcomes (Carnio et al., 2013; Uramoto and Tanaka, 2014). Therefore, identifying core regulators governing LUAD recurrence and elucidating the underlying mechanisms involved in the progression of LUAD would promote the development of effective strategies for diagnosis, treatment, and prognosis of this devastating disease.

As a systematic algorithm to identify tumor-specific indicators and predict cancer-related signaling pathways, the weighted gene co-expression network analysis (WGCNA) approach provides a comprehensive strategy to elucidate the interactions of pathogenic genes and to determine the correlation between gene networks and clinical traits (Langfelder and Horvath, 2008). With the help of WGCNA, genes were classified into different modules for functional prediction, while the most central genes could be further targeted as hub genes. Other than previous bioinformatics methodologies that mainly focus on individual genes, WGCNA implements methods for both weighted and unweighted correlation networks and displays the characteristics of biological systems more precisely (Barabási et al., 2011).

With the rapid advancement in recent years, WGCNA has been widely applied in the research of various cancer types (Liang et al., 2020; Yao et al., 2020; Liu et al., 2021). In terms of LUAD, DYNLRB2 and SPTBN1 were predicted to be tumor suppressors and may serve as biomarkers for LUAD patients (Zhu et al., 2021), while KIF11 was identified to be essential for LUAD cell proliferation and metastasis (Li et al., 2021). By utilizing WGCNA, long noncoding RNA (lncRNA) SVIl-AS1 was revealed to associate with chemoresistance in LUAD by acting as competing endogenous RNA (ceRNA) (Guo et al., 2021). Moreover, high-throughput data analysis by WGCNA indicates that SPP1 may be a key regulator in LUAD, which could be directly regulated by four miRNAs and indirectly regulated by 49 lncRNAs (Luo et al., 2021). However, comprehensive identification of biomarkers correlated with LUAD recurrence using WGCNA is yet to be investigated.

As an emerging technology, single-cell RNA-sequencing (scRNA-seq) is widely accepted as a powerful approach to study the heterogeneity of gene expression in individual cells (Suvà and Tirosh, 2019). By sequencing of LUAD cells harboring EGFR mutations, elevated expression of ELF3 was observed in advanced tumor cells, which promotes tumorigenesis through PI3K/AKT/NF-κB signaling pathway (He et al., 2021). Analysis of scRNA-seq data from LUAD samples manifested as subsolid nodules suggested that malignant cells in subsolid nodules undergo a strong metabolic reprogram and immune stress (Xing et al., 2021). Moreover, a multi-region scRNA-seq study revealed that LUAD exhibits pronounced intratumor cell heterogeneity within single sites and transcriptional lineage-plasticity programs (Sinjab et al., 2021).

Here in this study, WGCNA was constructed by using the GSE32863 dataset containing gene expression profiling data from 58 LUAD tumors and their matched normal adjacent lung tissue samples. Specific modules correlated with LUAD recurrence were identified, followed by hub gene prediction and functional analyses. Noteworthy, single-cell analysis was further applied to determine the expression of hub genes in different LUAD cell types, and the potential interactions between hub genes and therapeutic drugs were also investigated.

## Materials and Methods

### Data Acquisition and Preprocessing

The microarray dataset GSE32863 of human LUAD with patient clinical information was extracted from Gene Expression Omnibus (GEO) database (Selamat et al., 2012). GSE32863 dataset was obtained using Illumina HumanWG-6 v3.0 expression beadchip. It includes 58 LUAD samples, which were used to construct a co-expression network followed by extraction of hub genes. R package was used to annotate raw data, generate an expression matrix, and match probes with official gene symbols. The median absolute deviations (MADs) were ranked from the largest to smallest, and the expressions of the top 25% genes with the largest differences in the samples were extracted for in-depth analysis.

### Weighted Gene Co-Expression Network Analysis

We used the R package “WGCNA” to perform WGCNA on selected genes to find out the expression patterns between different genes (Langfelder and Horvath, 2008). Genes with similar expression patterns were regarded as a specific module and marked with a unique color. Subsequently, the correlation between these modules was calculated, and a heatmap was created to show the independence between each module. Then, a correlation analysis was performed to find modules related to clinical information. Modules closely related to tumor recurrence were chosen for further analysis.

### Gene Ontology and Pathway Enrichment Analysis

In order to understand the biological functions and signal pathways involved in the genes from the selected modules, differentially expressed genes (DEGs) in these modules were analyzed through the R package “clusterProfiler” (Yu et al., 2012). Then, another R package “ggplot2” was used to perform the top 10 enrichment terms of Gene Ontology (GO) analysis and Kyoto Encyclopedia of Genes and Genomes (KEGG) pathway analysis. Enriched GO terms and KEGG pathways were identified base on the cutoff criterion of *p* < 0.05.

### Protein–Protein Interaction of the Key Module Genes

For hub gene screening, genes from the selected modules were uploaded to the STRING database to build a protein–protein interaction (PPI) network (Szklarczyk et al., 2019). The interaction score >0.4 was defined as the threshold of the key genes in the PPI network. Then, a Cytoscape plug-in cytoHubba was used to extract the top 1% targets in these modules based on the degree method (Shannon et al., 2003).

### Identification and Validation of Real Hub Genes

Based on the cytoHubba analysis, the top 15 genes in the selected modules and the genes enriched in KEGG pathway were selected as candidate hub targets for further analysis. In this study, the Kaplan–Meier plotter (http://kmplot.com/analysis/) was used to draw the survival analyses of the candidate hub targets, while the Gene Expression Profiling Interactive Analysis (GEPIA) webserver (Tang et al., 2017) (http://gepia.cancer-pku.cn/) was used to verify the outcomes of survival analysis so as to screen out real hub genes. With the use of the Human Protein Atlas (http://www.proteinatlas.org) database, the real hub genes were further validated by immunohistochemistry (IHC). Moreover, Single Cell Portal (https://singlecell.broadinstitute.org/single_cell) was used to detect the expression of key targets in various cell types of lung cancer. In addition, the cBioPortal tool (http://www.cbioportal.org/; version: 2.2.0) was used to compare the genetic variations of the real hub genes in LUAD. Finally, the cBioPortal tool was also used to present the co-expression analysis of real hub genes.

### Gene Set Enrichment Analysis

In the validation set GSE116959, samples of LUAD were divided into low and high groups according to the expression level of the genes. To explore the potential function of real hub genes in LUAD, gene set enrichment analysis (GSEA) was conducted and mapped into KEGG pathway enrichment database. The terms with *p*-value <0.05 were chosen as the cutoff criteria.

### Construction of Transcription Factor–Real Hub Gene Network and Drug–Real Hub Gene Interaction

A regulating network on hub genes and LUAD was constructed by Cytoscape software. Then, the plugin iRegulon of Cytoscape was applied to forecast transcription factor (TF) regulation networks. In addition, Comparative Toxicogenomics Database (http://ctdbase.org/), a robust and publicly available database that aims to reveal how environmental exposures affect human health, was used to search for drugs for the hub genes. Chemicals supported by at least one database were selected as the potential drugs. Potential drug–key target interaction was constructed by Cytoscape. The final list only involves drugs that may interact with at least two key targets and have been reported to have an anticancer pharmacological activity.

## Results

### Identification of Key Modules Associated With Lung Adenocarcinoma Recurrence

**FIGURE 1:**
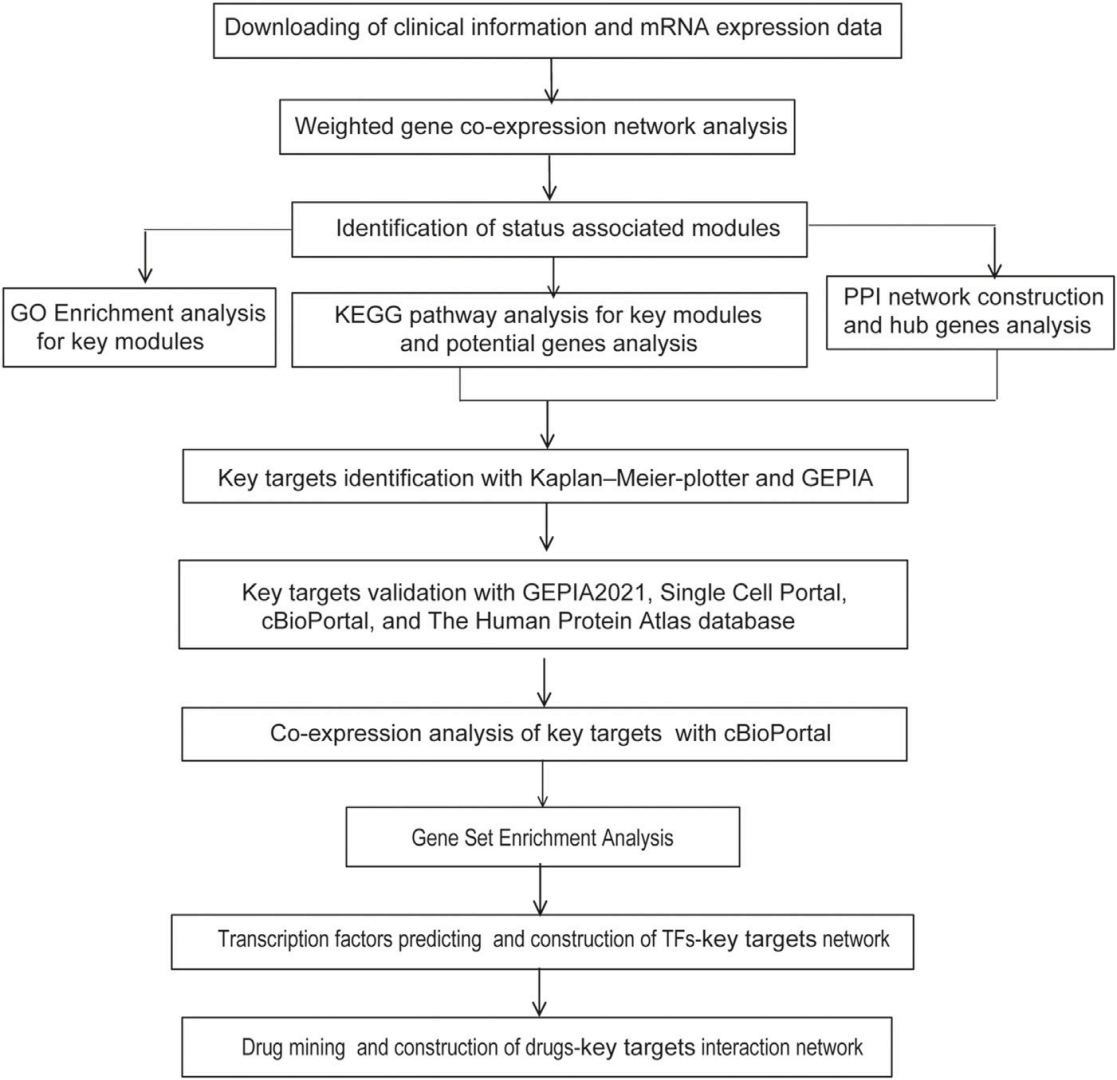
Experimental design and workflow of this study.

The flowchart of strategy in this study is summarized in Figure 1. GSE32863, a GEO dataset containing gene expression profiling data of 58 matched LUAD and non-tumor lung samples, was analyzed using the R package WGCNA. Clinical characteristics of tumor pathological stage, gender, recurrence, egfr, kras, lkb1, and smoking status of LUAD patients were denoted. After screening by MADs arranged from large to small, the expression of the top 25% genes (6,360 genes) with the greatest differences in samples were selected for further analyses.

**FIGURE 2:**
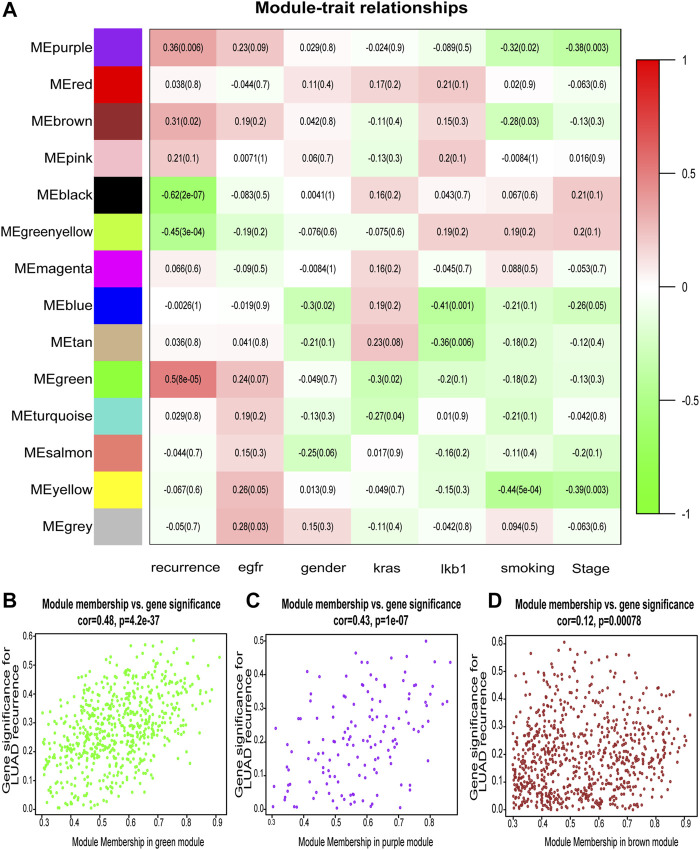
Identification of modules associated with the clinical features of lung adenocarcinoma (LUAD). **(A)** Heatmap to show the correlation between modules and clinical traits with LUAD. *p*-Values are shown in brackets. **(B–D)** Scatter plot analysis to show the association between Module membership and gene significance for LUAD recurrence in green **(B)**, purple **(C)**, and brown modules **(D)**.

After classification of DEGs with similar expression patterns into modules by average linkage clustering, 14 modules were finally identified by merging similar modules when the MedissThres was set at 0.25 (Figure 2A). Among them, module eigengenes of three modules (green, purple, and brown) were found to be more related to the tumor recurrence as others. Scatter plots further indicated positive correlations between members of these three modules and gene significance for LUAD recurrence (Figures 2B–D). Herein, the green, purple, and brown modules were chosen as key modules associated with LUAD recurrence for further investigation.

### Functional Analysis of Genes in Three Key Modules

**FIGURE 3:**
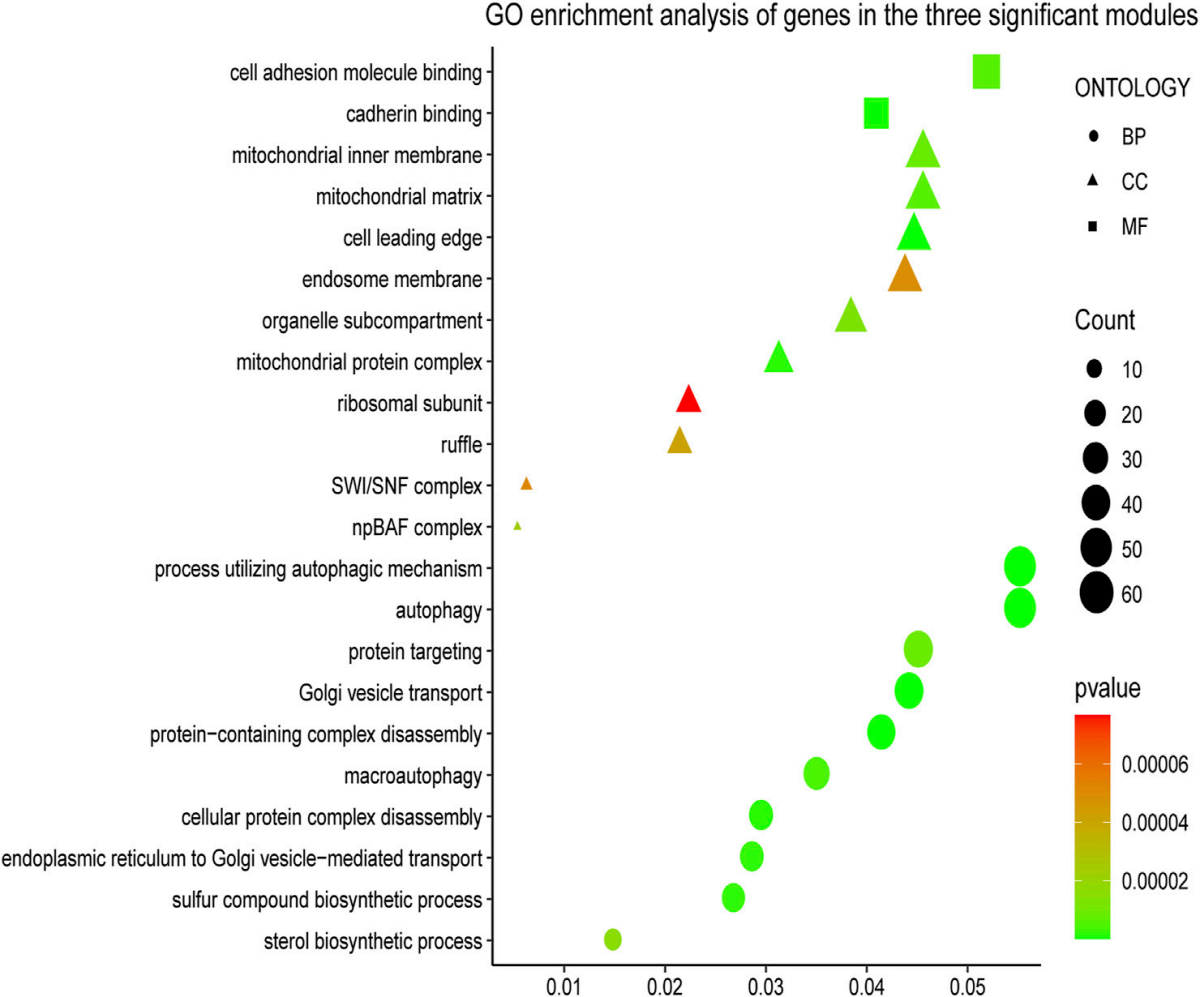
Gene Ontology (GO) enrichment analyses of gene members in three chosen modules. The colored geometry represents GO term enrichment, dots represent biological process (BP), triangles represent molecular function (CC), rectangles represent cellular component (MF), red indicates low enrichment, and green indicates high enrichment. The sizes of the geometries represent the number of genes in each GO category.

**FIGURE 4:**
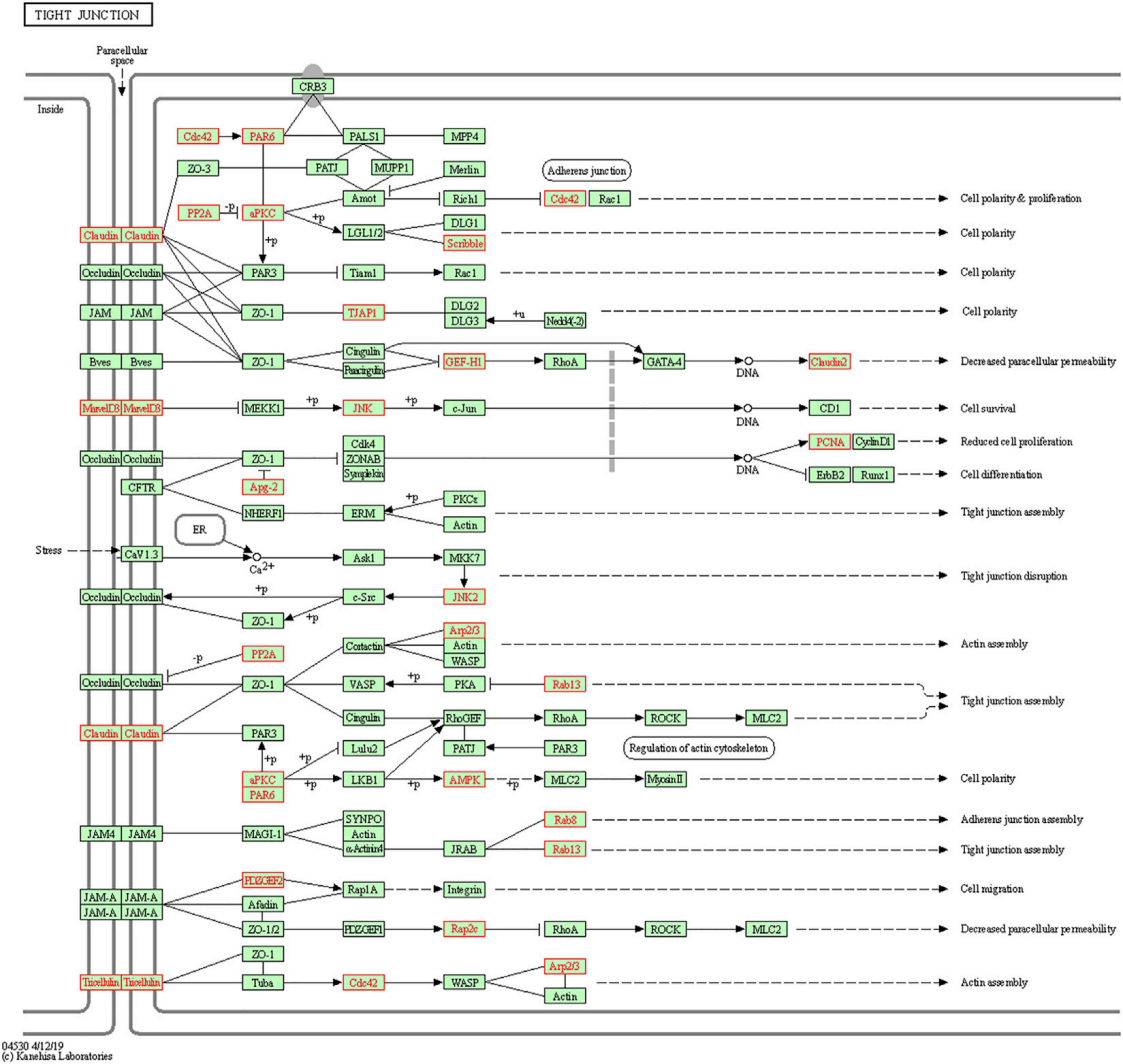
Kyoto Encyclopedia of Genes and Genomes (KEGG) pathway enrichment analyses. Genes in three modules (marked in red) could only be enriched in Tight Junction signaling pathway.

To understand the biological functions of genes in the selected modules, GO and KEGG pathway analyses were firstly applied. As depicted in Figure 3, genes in three key modules were expected to exert their functions in terms of cell adhesion molecule binding, endosome membrane, ribosomal subunit and autophagy, etc. Meanwhile, these genes could only be enriched in one single cellular pathway called Tight Junction signaling pathway (Figure 4 and Table 1).

**TABLE 1:** KEGG pathway analysis of the three significant modules genes related to LUAD recurrence.

KEGG	Description	*p*-Value	Genes	Count
hsa04530	Tight junction	1.35E−04	*MARVELD2*, *RAB8B*, *PCNA*, *PRKCI*, *ACTR3*, *RAP2C*, *PPP2R2A*, *CLDN7*, *CDC42*, *RAPGEF6*, *HSPA4*, *ARPC5*, *RAB13*, *PPP2R2B*, *CLDN14*, *MARVELD3*, *MAPK10*, *CLDN15*, *CLDN3*, *MAPK9*, *SCRIB*, *PARD6A*, *TJAP1*, *ARHGEF2*, *PRKAA1*	25

Note. KEGG, Kyoto Encyclopedia of Genes and Genomes; LUAD, lung adenocarcinoma.

**FIGURE 5:**
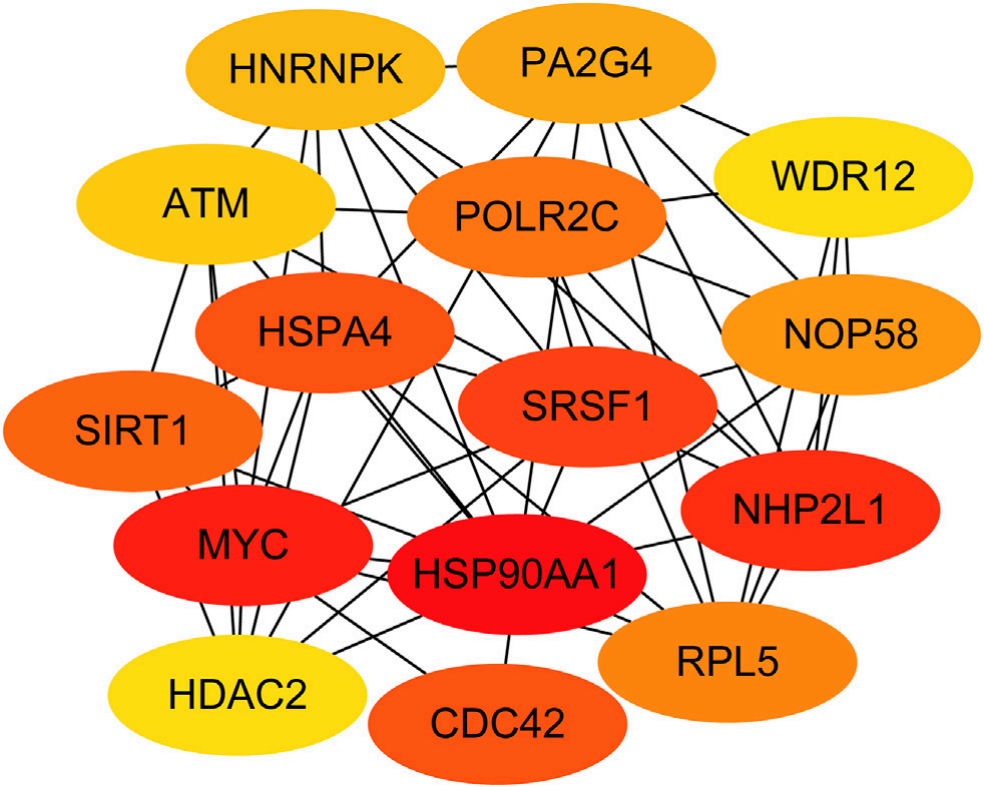
Identification of the top 15 genes from the protein–protein interaction (PPI) network of the three modules.

Next, genes in three key modules were extracted to establish a PPI network through STRING database and Cytoscape software. As a result, a total of 926 DEGs of the three key modules were mapped into the PPI network, including 1,426 nodes and 9,522 edges. In addition, the top 15 key targets within PPI network were selected with the help of cytoHubba plug-in in Cytoscape software based on rank of degree (Figure 5).

### Identification and Validation of Hub Genes for Lung Adenocarcinoma Recurrence

**FIGURE 6:**
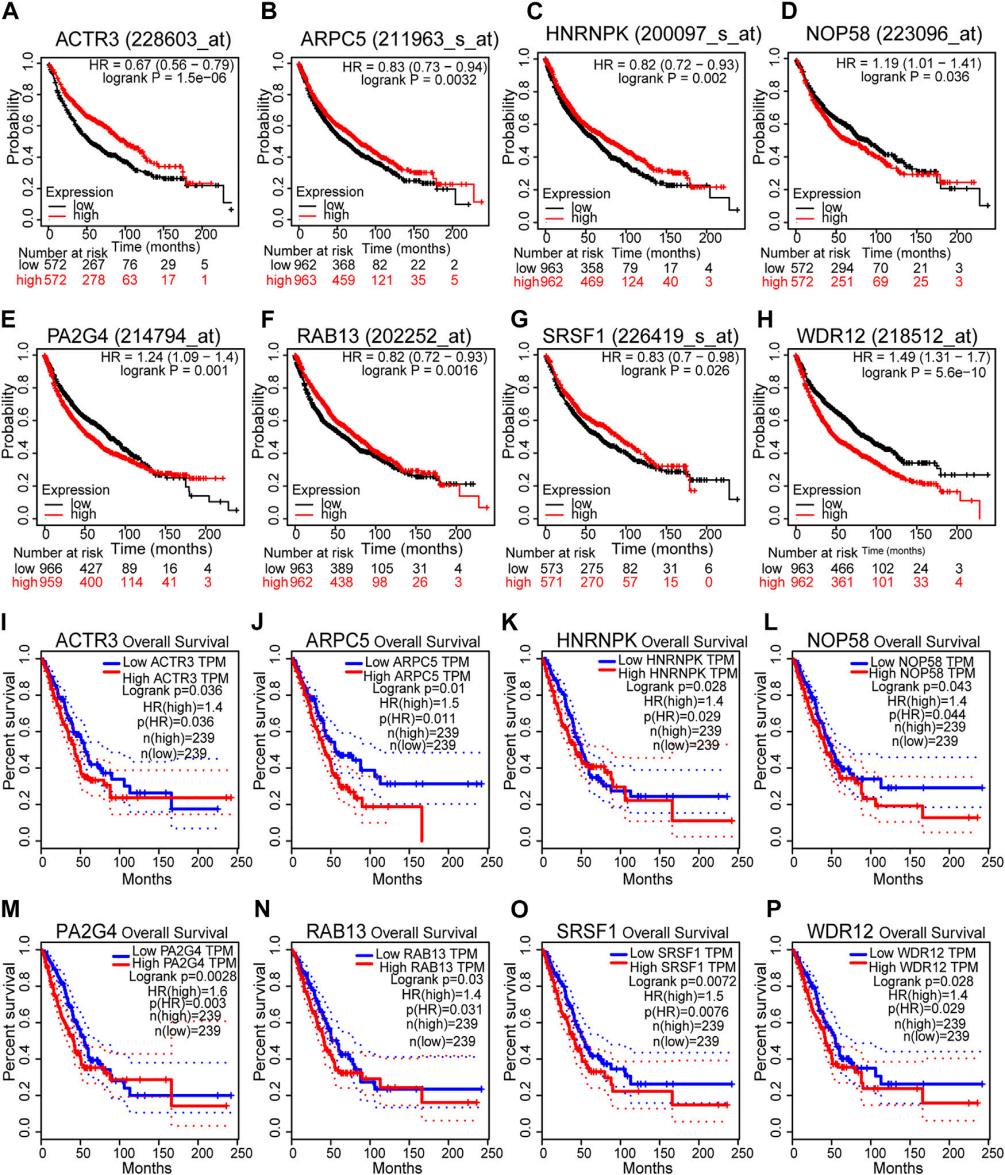
Survival analyses of the eight hub gene candidates identified. **(A–H)** Overall survival of the eight hub gene candidates in lung adenocarcinoma (LUAD) based on Kaplan–Meier plotter. **(I–P)** Overall survival of the eight hub gene candidates in LUAD based on the Gene Expression Profiling Interactive Analysis (GEPIA) database. *p* < 0.05 was considered to indicate a statistically significant difference. ACTR3, actin-related protein 3; ARPC5, actin-related protein 2/3 complex subunit 5; HNRNPK, heterogeneous nuclear ribonucleoprotein K; NOP58, NOP58 ribonucleoprotein; PA2G4, proliferation-associated 2G4; RAB13, RAB13 member RAS oncogene family; SRSF1, serine- and arginine-rich splicing factor 1; WDR12, WD repeat domain 12.

According to the results above, 40 genes (25 genes could be mapped into the KEGG pathway plus 15 key targets in PPI network) remained the potentials to be the hub genes for LUAD recurrence. For the purpose of scope reduction and effect evaluation, overall survival analysis was firstly performed on these candidate genes using the survival information of Kaplan–Meier plotter. After the first round of screening, only genes with significant impact on LUAD patient survival were further validated by survival analysis with GEPIA. As a result, a total of eight candidate hub genes (*ACTR3*, *ARPC5*, *RAB13*, *HNRNPK*, *PA2G4*, *WDR12*, *SRSF1*, and *NOP58*) were identified to be significantly correlated with LUAD patient survival with both survival analysis methods (Figure 6). Remarkably, all eight candidate genes were found to be correlated with poor LUAD patient survival through GEPIA, five of which (*ACTR3*, *ARPC5*, *RAB13*, *HNRNPK*, and *SRSF1*) were inversely revealed to be associated with favorable outcome using Kaplan–Meier plotter.

**FIGURE 7:**
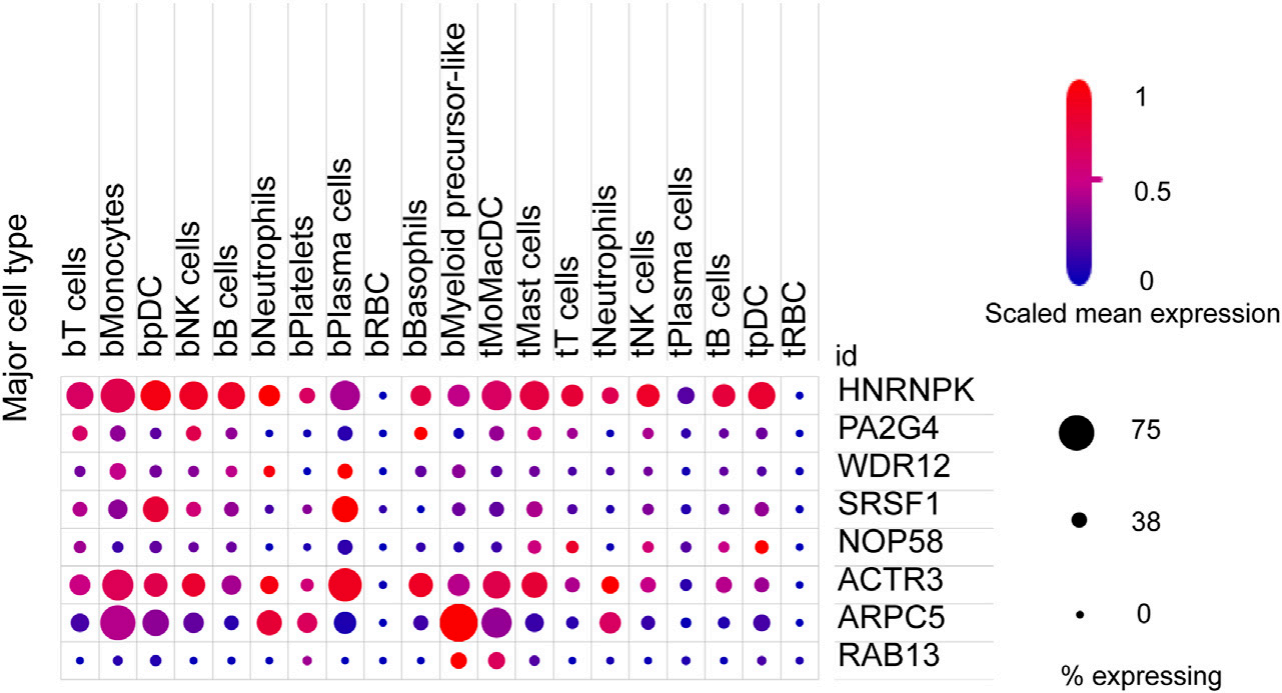
Expression of eight hub gene candidates in various clusters of human immune cells from non-small cell lung tumor (t) and peripheral blood (b). t, tumor; b, blood; DCs, dendritic cells; pDCs, plasmacytoid DCs; RBC, red blood cell. The size of dots represents the percentage of expression; red and blue represent the level of scaled mean expression.

**FIGURE 8:**
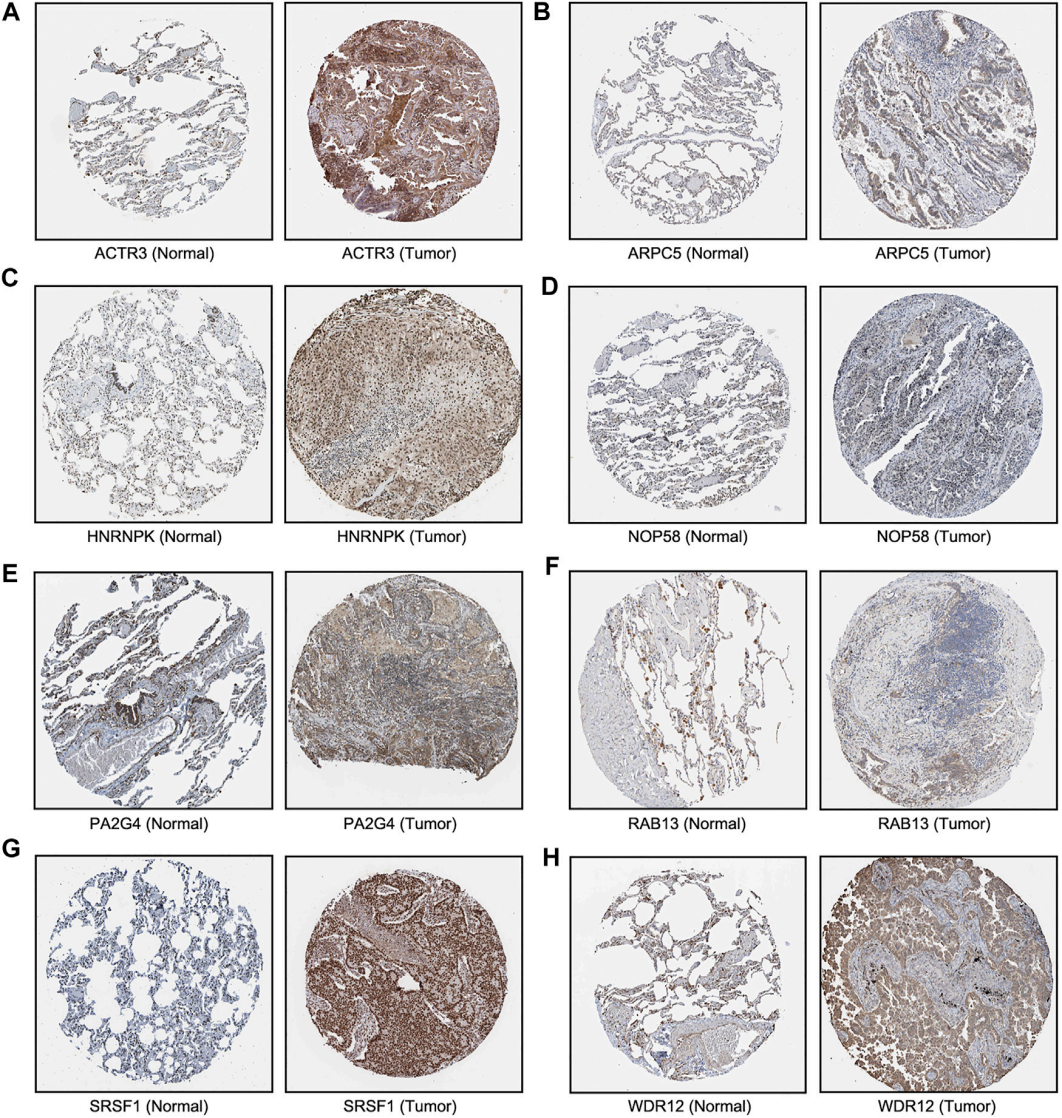
Immunohistochemical images of eight hub genes in lung adenocarcinoma (LUAD) recurrence. The protein levels of **(A–E)** in LUAD tissues were compared with normal lung tissues from the Human Protein Atlas database.

We next sought to investigate the expression of these candidate genes in LUAD. Firstly, comprehensive single-cell RNA sequencing data mapping myeloid populations in non-small cell lung tumor and peripheral blood was utilized to detect the expression of our candidate genes in different types of immune cells (Zilionis et al., 2019). As presented in Figure 7, candidate genes *ACTR3*, *ARPC5*, and *HNRNPK* were highly expressed in almost all types of lung cancer immune cells, while *RAB13*, *PA2G4*, and *WDR12* were found to be less abundant in the majority of myeloid populations in lung cancer. As compared with the rest of the hub genes, *ACTR3*, *ARPC5*, and *HNRNPK* are all characterized in regulation of actin polymerization (Yoo et al., 2006; Kopitar et al., 2019). Thus, the hyperactivation of these three genes in lung cancer cells may reflect the significance of actin polymerization during the development of cancer. Next, the distribution and expression of these eight hub gene candidates were checked using IHC staining data of both LUAD and adjacent non-tumor samples from The Human Protein Atlas database. Representative IHC images revealed elevated protein levels of HNRNPK, PA2G4, WDR12, SRSF1, and NOP58 in LUAD samples (Figure 8), suggesting the dysregulation of these genes during progression of LUAD.

**FIGURE 9:**
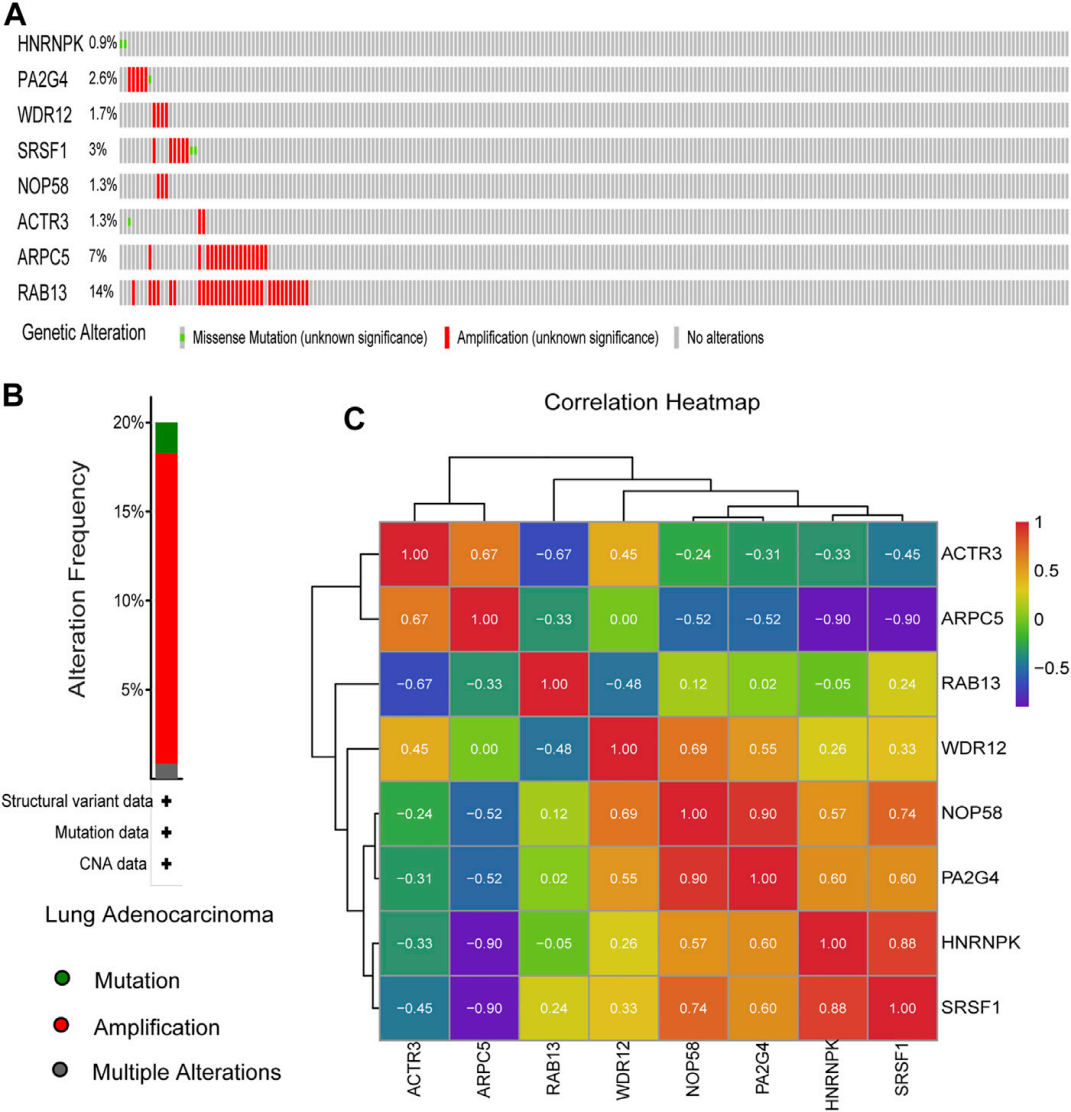
Genetic alteration information and co-expression analysis of the eight hub genes. **(A)** A visual summary across a set of lung adenocarcinoma (LUAD) (data from Lung Adenocarcinoma, The Cancer Genome Atlas (TCGA), *Nature* 2014) showed the genetic alterations connected with the eight hub genes, which were altered in 75 (31.8%) of 230 sequenced patients (230 in total). **(B)** An overview of changes in the eight hub genes from the genomics datasets of LUAD in TCGA database. Summary for lung adenocarcinoma: Gene altered in 20% of 230 cases, Mutation 1.74% (4 in 230 cases), Amplification 17.39% (40 in 230 cases), and Multiple Alterations 0.87% (2 in 230 cases). **(C)** The co-expression analysis of the eight hub genes using the 230 samples above based on cBioPortal database.

The genetic alterations of these candidate genes were then determined using cBioPortal. Interestingly, seven out of the eight genes were found to be amplificated in LUAD, while only half of them were reported to be partially mutated (Figures 9A,B). Gene co-expression analysis of the eight candidate genes was also performed using cBioPortal database. As shown in Figure 9C, highly positive co-expression was observed in pairs of PA2G4–NOP58, HNRNPK–SRSF1, and NOP58–SRSF1, while significantly negative co-expression was revealed in pairs of HNRNPK–ARPC5 and SRSF1–ARPC5.

**FIGURE 10:**
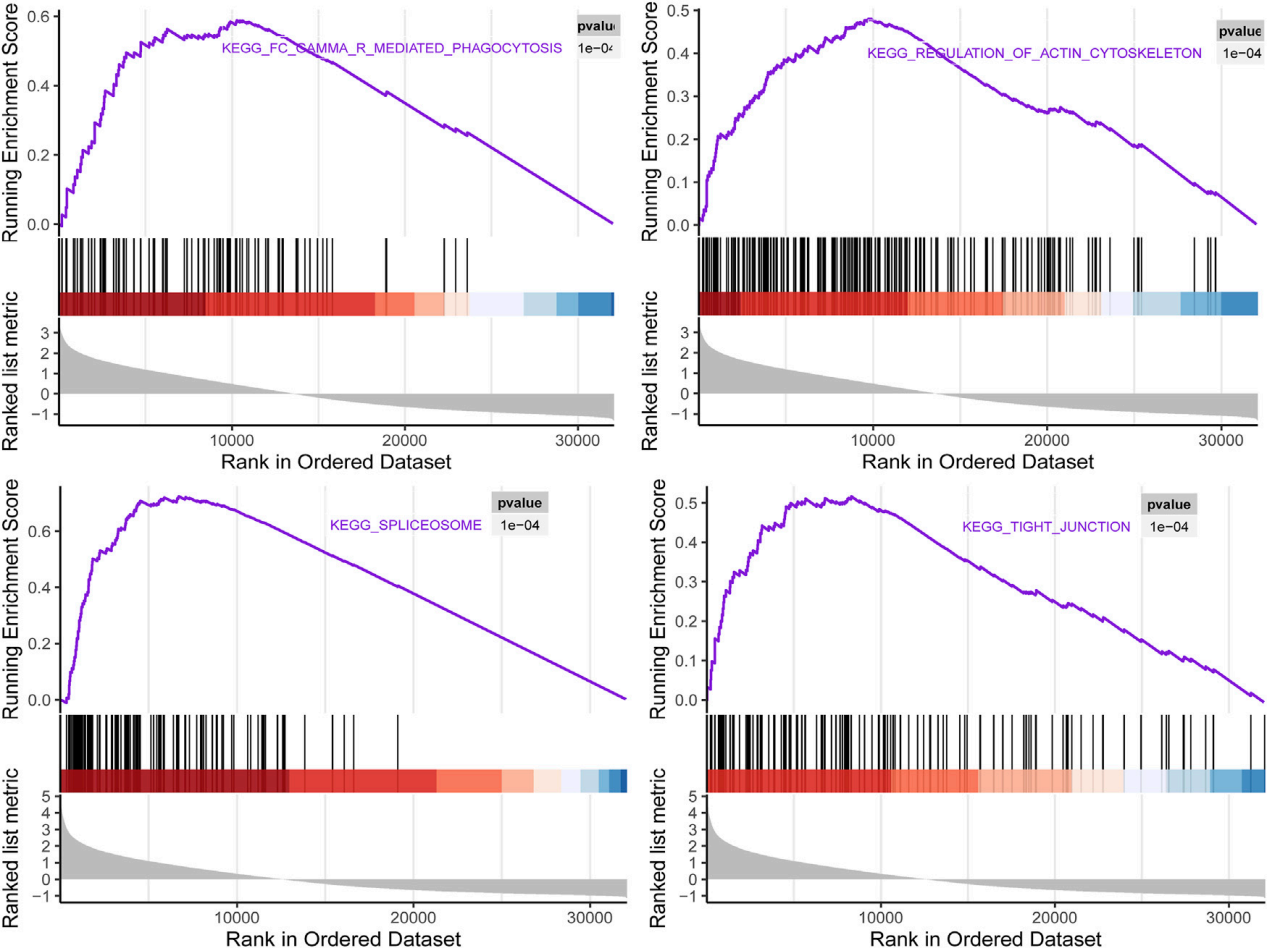
Gene set enrichment analysis (GSEA) using GSE116959 showed a positive enrichment of identified hub genes associated with four Kyoto Encyclopedia of Genes and Genomes(KEGG) pathways.

To investigate the biological characteristics of right candidate genes associated with LUAD recurrence, the GSEA assay was further applied. The results in Figure 10 showed that upregulation of these candidate genes was enriched in multiple cellular processed such as Fc gamma R-mediated phagocytosis, regulation of actin cytoskeleton, spliceosome, and tight junction.

In total, all the above findings indicated that all eight candidate genes may serve as the hub genes associated with tumor recurrence in LUAD.

### Constructions of Transcription Factor–Hub Gene Network and Drug–Hub Gene Interaction Network Associated With Lung Adenocarcinoma Recurrence

**FIGURE 11:**
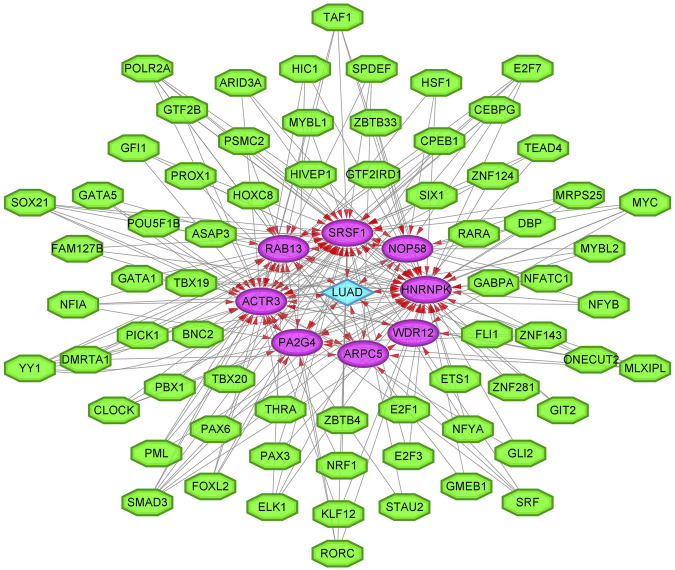
The transcriptional regulatory network of eight hub genes and TFs. TFs, transcription factors. A green hexagon node represents the TFs, a pink circular node represents hub genes, a light blue diamond node represents the lung adenocarcinoma (LUAD), and the interaction is represented by an arrow. The numbers of arrows in the networks demonstrate the contribution of 1 TF to the hub genes; and the higher the degree, the more central the nodes were within the network.

**FIGURE 12:**
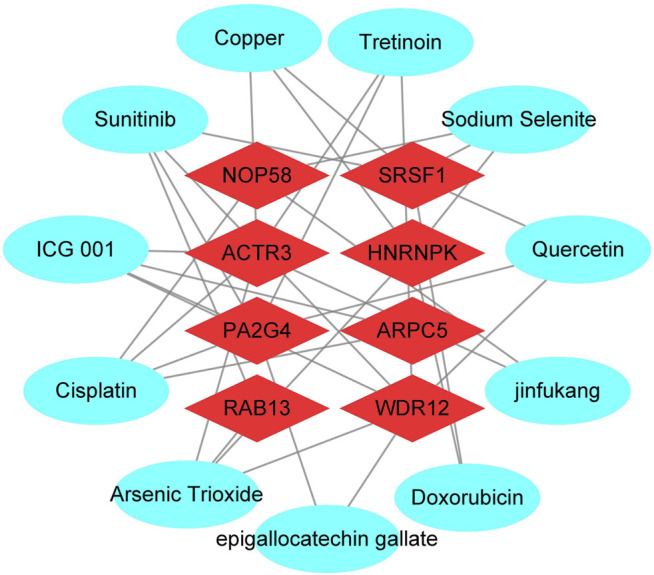
Interactions between drugs and the identified hub genes based on CTD database and literature validation. Light blue circular node represents drugs, red diamond node represents hub genes, their interaction is represented by line, and the degree value represents the number of targets acted on by the drug.

To enhance the significance of our study, we next sought to establish the transcriptional regulatory network of hub genes and TFs by a Cytoscape plug-in iRegulon. As revealed in Figure 11, a total of 57 TFs and eight hub genes were involved in this network, such as models of MYC–NOP58, MYC–WDR12, MYC–SRSF1, MYC–PA2G4, MYC–HNRNPK, PML–HNRNPK, TAF1–HNRNPK, YY1–HNRNPK, and TAF1–HNRNPK. To facilitate the future targeted drug screening, Comparative Toxicogenomics Database was used to search for drugs specialized in targeting the eight hub genes. Drugs that may interact with at least two hub genes and have been reported to have an anticancer pharmacological activity were selected. Finally, 11 drugs including arsenic trioxide, cisplatin, copper, ICG 001, Jinfukang, tretinoin, doxorubicin, sodium selenite, quercetin, sunitinib, and epigallocatechin gallate were discovered (Figure 12).

## Discussion

Despite the novel improvements in the diagnosis and treatment of LUAD in recent years, tumor recurrence remained to be the leading cause of treatment failure and mortality after surgery (Martini et al., 1995). With the help of WGCNA approach, a total of eight hub genes were screened out as LUAD recurrence-associated hub genes in the present study. To the best of our knowledge, most of them were firstly reported as key modulators of LUAD recurrence.

Among the identified hub genes, both *ACTR3* and *ARPC5* encode subunits of the human Arp2/3 protein complex, which has been implicated in the control of actin polymerization in cells (Abella et al., 2016). As compared with the less studied ACTR3, the potential of ARPC5 as a prognostic biomarker has been investigated in multiple cancer types such as hepatocellular carcinoma (Huang et al., 2021) and multiple myeloma (Xiong and Luo, 2018). In this study, we further confirmed the association between both Arp2/3 components and LUAD recurrence, which may extend the clinical relevance of these biomarkers into the prognosis of lung cancer.

As a member of Rab-associated G protein family, RAB13 controls the trafficking and cellular localization of a number of key modulators in cancer and thus affects the tumorigenesis (Ioannou and McPherson, 2016). Based on The Cancer Genome Atlas (TCGA) database, elevated expression of RAB13 has been observed in the majority of cancers and is inversely correlated with patient prognosis. Moreover, by analyzing published microarray data for NCI-60 cancer cells, RAB13 was further discovered as a target gene relevant to radiosensitivity (Kim et al., 2012). Here, we observed significant amplification of RAB13 in LUAD sample, which deserves to be further investigated to unveil its role in the carcinogenesis of LUAD.

Interestingly, five out of the eight hub genes identified in the current study (*HNRNPK*, *PA2G4*, *WDR12*, *SRSF1*, and *NOP58*) are mainly located in the nucleus and are involved in functions of rRNA processing, RNA splicing, and ribosome biogenesis. For instance, HNRNPK is a component of the hnRNP complex and a highly conserved RNA- and DNA-binding protein (Pinol-Roma et al., 1988). Downregulation of HNRNPK in human LUAD cell line significantly reduced the formation of metastatic lung tumor nodules in mice, suggesting its role in lung metastasis (Li et al., 2019). Another example is NOP58, which forms box C/D small nucleolar ribonucleoprotein (snoRNP) with three other protein members to exert its functions in rRNA methylation and ribosome biogenesis (Shubina et al., 2016). Dysregulation of snoRNP members including NOP58 has been widely reported in cancer, which directly contributes to the overactivated ribosome biogenesis during cancer progression (Wu et al., 2020; Yi et al., 2021). Therefore, it is speculated that hyperactivation of ribosome biogenesis, which can be initiated by these hub genes, may play a crucial role in LUAD recurrence.

In addition, transcription factors (TFs) that regulate hub genes have been predicted, including MYC (Massó-Vallés et al., 2020), PML (Kuo et al., 2014), and YY1 (Lin et al., 2020). It has been reported that they were potential therapeutic targets for lung cancer and were closely related to lung cancer metastasis and recurrence. Finally, through CTD database and literature mining, it was discovered that arsenic trioxide (Huang and Zeng, 2019), Jinfukang (Que et al., 2021), sunitinib (Park and Kim, 2020), and other drugs reported to treat lung cancer could act on these eight hub genes.

In general, our current study aimed to find hub genes that might be correlated with the recurrence of LUAD using WGCNA approach. A total of eight genes including *ACTR3*, *ARPC5*, *RAB13*, *HNRNPK*, *PA2G4*, *WDR12*, *SRSF1*, and *NOP58* were identified and further validated using multiple bioinformatics tools. It is anticipated that these hub genes could serve as biomarkers or therapeutic targets for LUAD treatment.

## Data Availability

The datasets presented in this study can be found in online repositories. The names of the repository/repositories and accession number(s) can be found in the article/Supplementary Material.
